# Musculoskeletal pain associated with common mental disorders among bus terminal workers in Brazil

**DOI:** 10.47626/1679-4435-2024-1301

**Published:** 2025-01-31

**Authors:** Sabrina Gabriele Maia Oliveira Rocha, Hermano Alexandre Lima Rocha, Sâmia Graciele Maia Oliveira Giacomini, Anamerinda de Oliveira Diaz, Luciano Lima Correia

**Affiliations:** 1 Department of Community Health, Universidade Federal do Ceará, Fortaleza, CE, Brazil.

**Keywords:** occupational health, common mental disorders, musculoskeletal pain, saúde ocupacional, transtornos mentais comuns, dor musculoesquelética

## Abstract

**Introduction:**

Common mental disorders affect almost one billion people worldwide and are among the main causes of work absenteeism, representing a high cost for society.

**Objectives:**

We aimed to identify factors associated with symptoms of common mental disorders among workers in urban bus terminals in Fortaleza, Brazil.

**Methods:**

This analytical cross-sectional study was based on data from periodical and pre-admission medical examinations performed in 2022. We investigated mental disorder symptoms with the Depression Anxiety & Stress Scales 21, correlating associated factors through multivariate analysis.

**Results:**

The prevalence of stress and anxiety was about 11%, while that of depression was 8.3%. Self-reported leg pain was associated with depression, anxiety, and stress. Smoking was associated with depression (adjusted odds ratio 4.96; p = 0.002) and stress (adjusted odds ratio 4.08; p = 0.003). Back pain was associated with stress (adjusted odds ratio 2.26; p = 0.018).

**Conclusions:**

To develop initiatives to reduce symptoms of common mental disorders and absenteeism, common mental disorders in workers should be assessed jointly with associated factors, such as chronic pain, smoking, and social factors.

## Abstract (pt)

**Introdução:**

Os transtornos mentais comuns afetam quase um bilhão de pessoas no mundo e estão entre as principais causas de absenteísmo no trabalho, representando um alto custo para a sociedade.

**Objetivos:**

Este estudo visa identificar os fatores associados aos sintomas dos transtornos mentais comuns em trabalhadores dos terminais rodoviários de Fortaleza, Brasil.

**Métodos:**

Trata-se de um estudo transversal analítico, com dados de exames periódicos admissionais e demissionais de 2022, de trabalhadores ativos de terminais rodoviários de Fortaleza. Investiga transtornos mentais através da Escala de Ansiedade, Depressão e Estresse 21 adaptada em português, correlacionando-os com dados demográficos, ocupacionais, comorbidades e hábitos, por análise multivariada.

**Resultados:**

Encontramos prevalência de 11% de estresse e ansiedade e 8,3% de depressão nesses trabalhadores. Observamos que a dor autorreferida em membros inferiores estava associada com depressão, ansiedade e estresse. O tabagismo estava associado à depressão (razão de chances ajustada 4,96; p = 0,002) e ao estresse (razão de chances ajustada 4,08; p = 0,003). A dor lombar estava associada a sintomas de estresse (razão de chances ajustada 2,26; p = 0,018). Morar com menos de três pessoas estava associado a sintomas de ansiedade e depressão; não ter companheiro estável estava associado à depressão.

**Conclusões:**

A avaliação de transtornos mentais comuns em trabalhadores deve ser acompanhada da avaliação de fatores associados, como dores crônicas, tabagismo e fatores sociais, com o objetivo de gerar ações para reduzir os sintomas de transtornos mentais comuns e o absenteísmo.

## INTRODUCTION

Almost one billion people worldwide are affected by common mental disorders (CMD), of which depression and anxiety are the most common.^1^ These disorders are among the main causes of work absenteeism,^2^ resulting in high costs for society.^3^ CMD, a group of disorders commonly found in the community and primary health care services, include mood and somatic disorders, such as anxious and/or depressive mood. They result in dysfunction in everyday activities, even without affecting cognition or perception.^4^

According to a systematic review based on data from 63 countries, approximately one in four individuals has experienced some type of mental disorder at some point in their lives, and approximately one in five people has had a CMD in the last 12 months.^5^ The World Health Organization estimates that over 300 million people worldwide have a depressive disorder and over 200 million have an anxiety disorder.^1^ The prevalence of mental disorders has been gradually increasing in Brazil in recent decades, resulting in the highest anxiety prevalence in the world. It is estimated that almost 19 million Brazilians, about 9.3% of the population, have anxiety disorders,^1^ the most prevalent of which is generalized anxiety disorder, followed by panic disorder and post-traumatic stress disorder. Phobic disorders are also increasingly common among the general population.^6^ In addition, the 2019 Brazilian National Health Survey estimated that 10.2% of people over 18 years of age have been diagnosed with depression by a mental health professional.^7^

The genesis of these mental disorders is multifactorial, with no single specific cause, but rather a complex interaction of several factors that can vary from person to person. Genetic factors, family history, and biological factors, such as chemical imbalances in the brain, may predispose individuals to these disorders.^8^ In addition, associating socioeconomic and environmental factors with stressful situations and traumatic events, such as loss, conflict, and violence, can trigger depression and anxiety crises.^9^

Other factors are also correlated with mental disorders. Unhealthy lifestyle habits such as addictions, smoking, and substance abuse, are associated as both causes and consequences of mental disorders.^10^ Certain pathologies and comorbidities are also associated with mental disorders, such as sleep disorders,^11^ chronic disease, polypharmacy, and acute or chronic pain,^12^ which can trigger, be triggered by, or prolong mental disorders.^13^ Joint pain and chronic muscle pain, including low back pain, which are highly prevalent worldwide,^14^ are also closely associated with mental disorders, whether as a cause^15^ or a consequence.^16^ Joint and muscle pain is a common complaint among workers and is one of the main causes of absenteeism (for the overall number of medical excuses as well as the number days missed from work).^17^

A recent Brazilian study based on data from the National Social Security Institute found that diseases of the musculoskeletal system/connective tissue (including muscle pain in various regions, such as the lower back) and mental/behavioral disorders were the second and third causes, respectively, of work absences longer than 15 days.^2^ The profile of work absences due to mental disorders is changing, with a lower overall number of absences but an increase in the total number of days per absence. Due to their high prevalence and negative repercussions, CMD represent a high cost to society.^3^

The general increase in individuals with mental disorders is a complex and multifaceted phenomenon. However, because mental balance is linked to economic balance in society, it is imperative to determine the factors associated with CMD symptoms to develop strategies for reducing the burden of illness and work absences and to improve worker quality of life and productivity. Therefore, this study aimed to identify demographic, occupational, and comorbid factors associated with CMD symptoms among of bus terminal workers in metropolitan Fortaleza, Brazil.

## METHODS

### STUDY DESIGN AND SAMPLE

This analytical cross-sectional study was conducted in a company that manages bus terminals in Fortaleza, Brazil’s fifth largest city (population > 2.7 million). Fortaleza has nine urban bus terminals, in addition to two express corridors, and three interurban bus terminals, through which more than 600,000 passengers travel per day. The company has an active staff of around 688 formally contracted employees.

The study population consisted of workers who perform bus and passenger control (terminal agents), maintenance, cleaning, terminal telemonitoring, or administrative activities. Data were used from active employees in 2022 who underwent either periodical or pre-admission assessment, with duplicate assessment data being excluded. Inactive employees (e.g., on leave), trainees/probationary employees, and workers who were hired but did not join the active workforce were excluded from the analysis.

### DATA COLLECTION

Data were collected by an occupational physician through a structured, self-report questionnaire during periodical or pre-admission assessments. Weight and height measurements were taken. The data were entered into a spreadsheet after masking through Google Forms.

### VARIABLES

#### Outcome variables

CMD symptoms were investigated using the Portuguese version of the Depression, Anxiety, and Stress Scale-21, whose Cronbach’s alpha (internal consistency) was 0.92 for the depression subscale, 0.90 for the stress subscale, and 0.86 for the anxiety subscale.^18^ This self-report instrument includes 21 questions on feelings in the last week, which are scored according to a four-point Likert-type scale, ranging from 0 (does not apply to me) to 3 (applies very much to me). Questions 1, 6, 8, 11, 12, 14, and 18 are the stress subscale. Questions 2, 4, 7, 9, 15, 19, and 20 are the anxiety subscale. Questions 3, 5, 10, 13, 16, 17, and 21 are the depression subscale. To determine the final score, the subscale are summed and multiplied by two to correspond to the original 42-question scale. Stress symptom scores are classified as: 0-10 = normal, 11-18 = mild, 19-26 = moderate, 27-34 = severe, and 35-42 = extremely severe. Anxiety symptom scores are classified as: 0-6 = normal, 7-9 = mild, 10-14 = moderate, 15-19 = severe, and 20-42 = extremely severe. Depression symptom scores are classified as: 0-9 = normal, 10-12 = mild, 13-20 = moderate, 21-27 = severe, and 28-42 = extremely severe.^19^ Dichotomous variables were used in the analysis, i.e., having symptoms of stress, anxiety, and depression or not.

#### Independent variables

We collected data on demographics (age, sex, marital status, religion, and the number of people living in the worker’s home), occupation (job position – siting or standing; sector – operational, maintenance, cleaning, or administrative; shift – day or night; and workday length [hours]), comorbidities (chronic non-transmissible diseases, such as diabetes, hypertension, overweight, cancer, musculoskeletal pain), and lifestyle habits (smoking, alcohol use, physical activity, sleep quality). Body mass index was calculated from weight and height measurements taken during the medical evaluation.

### STATISTICAL ANALYSIS

The data were analyzed in IBM SPSS Statistics 17. Frequencies and measures of central tendency were determined, and the normality of numerical variables was determined through the Kolmogorov-Smirnov test. The chi-square and Mann-Whitney tests were used to determine the association between the independent variables and the outcomes. To control for confounding, multivariate logistic regression of factors with p < 0.05 was performed. Missing data were excluded.

### ETHICAL ASPECTS

This study was approved by the Centro Universitário Christus Research Ethics Commitee (decision 4,668,150). After the objectives and risks of the study were described, those who agreed to participate provided writen informed consent.

## RESULTS

We analyzed data from 525 employees (77.8% of the active work force). One-third of the employees work the night shift and almost 85% work 12-hour days. Less than 10% work in a siting position, almost 35% work in a standing position, and more than 50% change positions during their shift (Table 1). The prevalence of stress symptoms was 11.1%, which were mainly mild (60.7% of the cases). The prevalence of anxiety symptoms was 11.2%, which were mainly moderate, although 26.3% of the cases involved extreme symptoms. The prevalence of depression symptoms was 8.3%, which were mainly moderate. The sample consisted of 41% women, and 26.7% were under 30 years of age. More than 30% reported living with less than three people, and 5.2% reported smoking. More than 20% reported back pain, 8.6% reported arm pain, and 18.4% reported leg pain (Table 1).

**Table 1:** The prevalence of common mental disorders, characteristics, and comorbidities among bus terminals workers, Fortaleza, Brazil 2022

	n	%
Stress (yes)	56	11.1
Stress categories		
Normal	450	88.9
Mild	34	6.7
Moderate	11	2.2
Severe	10	2.0
Extreme	1	0.2
Anxiety (yes)	57	11.2
Anxiety categories		
Normal	450	88.8
Mild	17	3.4
Moderate	18	3.6
Severe	7	1.4
Extreme	15	3.0
Depression (yes)	42	8.3
Depression categories		
Normal	465	91.7
Mild	8	1.6
Moderate	19	3.7
Severe	7	1.4
Extreme	8	1.6
Sex		
Female	215	41.0
Male	309	59.0
Age (years)		
< 30	140	26.7
> 30	384	73.3
Education (years)		
≤ 9	66	13.0
> 9	441	87.0
Partner (no)	257	49.4
Number of people in the home		
< 3	159	31.0
> 3	354	69.0
Shift		
Diurnal	348	66.7
Nocturnal	174	33.3
Workday (hours)		
12	441	84.2
< 12	83	15.8
Work posture		
Sitting	47	9.4
Standing	174	34.8
Alternating	279	55.8
Disabled (yes)	25	4.8
Diabetes (yes)	25	4.8
Hypertension (yes)	46	8.9
Overweight (yes)	375	72.5
Back pain (yes)	111	21.3
Arm pain (yes)	45	8.6
Leg pain (yes)	96	18.4
Smoker (yes)	27	5.2
Alcohol user (yes)	110	21.4
Physical activity (no)	198	38.0

### FACTORS ASSOCIATED WITH STRESS SYMPTOMS

The prevalence of stress was higher among workers who reported pain in their back, arms, and legs (p < 0.001; p = 0.006; p < 0.001, respectively) than among those who did not. This was also the case for workers with hypertension (p = 0.041) and smokers (p < 0.001) compared to normotensive individuals and non-smokers. Administrative workers, those with < 12-hour workdays (p = 0.046), and those who spend more time siting (p = 0.037), were more likely to have stress symptoms than factory workers, those who have a 12-hour workday, and those who spend more time standing or changing postures (Table 2).

**Table 2:** Factors associated with common mental disorders – bivariate analysis

	Stress				Anxiety				Depression			
	N	n	%	p-value	N	n	%	p-value	N	n	%	p-value
Demographics												
Sex				0.464				0.027				0.738
Female	203	25	12.3		207	31	15.0		205	18	8.8	
Male	303	31	10.2		300	26	8.7		302	24	7.9	
Age (years)				0.081				0.390				0.592
< 30	140	21	15.0		136	18	13.2		139	13	9.4	
> 30	366	35	9.6		371	39	10.5		368	29	7.9	
Education (years)				0.676				0.611				0.834
≤ 9	61	6	9.8		63	6	9.5		65	6	9.2	
> 9	429	50	11.7		427	50	11.7		426	36	8.5	
Partner				0.337				0.054				0.033
No	248	31	12.5		249	35	14.1		244	27	11.1	
Ye s	255	25	9.8		255	22	8.6		259	15	5.8	
Number of people in the home				0.053				0.011				0.013
< 3	151	23	15.2		154	26	16.9		150	19	12.7	
> 3	344	32	9.3		343	31	9.0		346	21	6.1	
Work characteristics												
Shift				0.164				0.365				0.534
Nocturnal	168	14	8.3		169	16	9.5		170	12	7 .1	
Diurnal	337	42	12.5		337	41	12.2		335	29	8.7	
Workday (hours)				0.046				0.967				0.928
12	426	42	9.9		426	48	11.3		425	35	8.2	
< 12	80	14	1 7.5		81	9	11.1		82	7	8.5	
Work posture				0.037				0.069				0.150
Sitting	44	10	22.7		41	9	22.0		46	5	10.9	
Standing	168	16	9.5		166	18	10.8		167	19	11.4	
Alternating	272	28	10.3		276	27	9.8		270	17	6.3	
Comorbidities												
Hypertension				0.041				0.009				0.836
Yes	39	8	20.5		44	10	22.7		41	3	7.3	
No	462	46	10.0		458	45	9.8		461	38	8.2	
Overweight				0.397				0.436				0.206
Yes	360	43	11.9		362	43	11.9		360	33	9.2	
No	140	13	9.3		138	13	9.4		140	8	5.7	
Back pain				< 0.001				0.001				0.001
Yes	104	25	24.0		102	21	20.6		106	17	16.0	
No	400	31	7.8		403	35	8.7		399	25	6.3	
Arm pain				0.006				0.012				0.321
Yes	42	10	23.8		44	10	22.7		41	5	12.2	
No	462	46	10.0		462	47	10.2		463	36	7.8	
Leg pain				< 0.001				0.002				0.002
Yes	90	25	2 7.8		87	18	20.7		91	15	16.5	
No	414	31	7.5		417	39	9.4		413	27	6.5	
Habits												
Smoking				< 0.001				0.040				< 0.001
Yes	27	10	3 7.0		25	6	24.0		27	8	29.6	
No	476	45	9.5		479	51	10.6		477	34	7 .1	
Physical activity				0.380				0.134				0.184
No	189	24	12.7		188	26	13.8		192	20	10.4	
Yes	315	32	10.2		316	30	9.5		312	22	7.1	

### FACTORS ASSOCIATED WITH ANXIETY SYMPTOMS

Pain in the back, arms, and legs were associated with anxiety symptoms (p = 0.001; p = 0.012; p = 0.002, respectively), as were hypertension and smoking (p = 0.009; p = 0.040, respectively). The prevalence of anxiety was higher among women than men (p = 0.027). We also found a higher prevalence of anxiety among employees who reported living with < 3 people (p = 0.011) than among those who lived with = 3 people (Table 2).

### FACTORS ASSOCIATED WITH DEPRESSION SYMPTOMS

Factors associated with a higher prevalence of depression symptoms included back pain (p = 0.001), leg pain (p = 0.002), smoking (p < 0.001) and living with < 3 people (p = 0.013). Those without a partner had a higher prevalence of depression (p = 0.033) than those who did (Table 2).

Leg pain increased the odds of stress symptoms (adjusted odds ratio [OR_a_]= 2.82; 95%CI 1.42-5.57; p = 0.003), anxiety symptoms (OR_a_= 2.46; 95%CI 1.28-4.71; p = 0.007), and depression symptoms (OR_a_= 2.23; 95%CI 1.00-4.96; p = 0.049). Those who reported back pain were 2.26 times more likely to have stress symptoms (95%CI 1.15-4.42; p = 0.018). Smokers were 4.08 (95%CI 1.61-10.36; p = 0.003) and 4.96 (95%CI 1.82-13.56; p = 0.002) more likely to have stress and depression symptoms, respectively. Living with < 3 people increased the odds of anxiety symptoms by 1.97 times (95%CI 1.09-3.53; p = 0.024) and the odds of depression symptoms by 2.06 times (95%CI 1.03-4.15; p = 0.042) compared to those who lived with = 3 people. The odds of depression symptoms were 2.42 times higher (95%CI 1.17-4.98; p = 0.018) among those who did not have a partner than among those who did. Hypertensive workers were 2.67 (95%CI 1.22-5.84; p = 0.014) more likely to have anxiety symptoms than normotensive workers (Table 3).

**Table 3:** Factors that remained associated with common mental disorders after multivariate analysis

	OR_a_	95%CI	p-value
Stress*			
Leg pain	2.82	1.42-5.57	0.003
Back pain	2.26	1.15-4.42	0.018
Smoking	4.08	1.61-10.36	0.003
Arm pain	1.11	0.44-2.78	0.828
Hypertension	1.97	0.81-4.82	0.138
12-hour of work	1.80	0.88-3.67	0.106
Anxiety†			
Leg pain	2.46	1.28-4.71	0.007
< 3 people in the home	1.97	1.09-3.53	0.024
Hypertension	2.67	1.22-5.84	0.014
Back pain	1.70	0.86-3.34	0.124
Arm pain	1.37	0.56-3.39	0.492
Female	1.64	0.91-2.96	0.101
Smoking	2 .10	0.69-6.37	0.189
Depression‡			
Leg pain	2.23	1.00-4.96	0.049
No partner	2.42	1.17-4.98	0.017
< 3 people in the home	2.06	1.03-4.15	0.042
Smoker	4.96	1.82-13.56	0.002
Back pain	2 .12	0.98-4.61	0.058

OR_a_ = adjusted odds ratio.

* Model variables: work hours, hypertension, back pain, arm pain, leg pain, and smoking.

† Model variables: sex, number of people in the home, hypertension, back pain, arm pain, leg pain, and smoking.

‡ Model variables: having a partner, number of people in the home, back pain, leg pain, and smoking.

## DISCUSSION

In this population of bus terminal workers in Fortaleza, Brazil, the prevalence of stress and anxiety symptoms was about 11%, while that of depression symptoms was 8.3%. Self-reported leg pain was independently associated with depression, anxiety, and stress symptoms. Back pain was associated with stress symptoms. Smoking was associated with both depression and stress symptoms. Living with < 3 people was associated with anxiety and depression symptoms, and not having a partner was associated with depression symptoms.

### COMMON MENTAL DISORDERS AND CHRONIC PAIN

Leg pain more than doubled the likelihood of depression, anxiety, and stress symptoms, while back pain more than doubled the likelihood of stress symptoms. The prevalence of chronic pain is high worldwide. According to a 2019 Brazilian Ministry of Health survey, about 37.6% of adults reported chronic or persistent (> 3 months) pain. Chronic pain can be a somatic symptom triggered by mental disorders. Neurotransmiter imbalance, such as low serotonin and norepinephrine levels, may be responsible for these symptoms.^20^ Coexisting conditions can aggravate both symptom types.^21^

Due to the prevalence and interrelation between pain and CMD, it is expected that they will be associated in the general population. However, most studies have investigated chronic pain in general terms^12,13^ or low back pain.^15,16^ Brazilian studies have found an association between low back pain and CMD,^22^ including a study with patients assisted in a community health seting.^23^ However, most Brazilian studies have assessed the general population or health sector workers; they rarely address workers in the service sector. We found an association between CMD and leg pain, which very few other studies have reported. Further investigation is needed to determine the factors involved in this association and the effects of somatization. There is also no practical approach for investigating such an association in primary or preventive health services.^23^ Thus, mental health care should be integrated with that of physical conditions.^24^

### SMOKING AND COMMON MENTAL DISORDERS

Smokers were almost five times more likely to have depressive symptoms and were four times more likely to have stress symptoms. This association has been thoroughly documented in the literature, with smoking reported as both a prior and subsequent factor to CMD onset,^25^ although less expressively than in our study. Population studies indicate that smoking leads to stress and depression symptoms^26^ and reduces the age of onset of depressive symptoms. However, the mechanisms of this association have not been well established. Other studies argue that the negative effects of CMD lead to smoking.^27^ Regardless of whether the relationship is cause, effect, or bidirectional, smoking must be addressed in CMD management.

### SOCIAL FACTORS

Workers who reported living with < 3 people were 1.97 times more likely to have anxiety symptoms and were 2.06 times more likely to have depression symptoms. Living without a partner increased the likelihood of depression symptoms by 2.42 times. A lack of emotional and financial support may be involved in this relationship.

There is evidence that the relationship between depression and marital status is not direct and that it can be modified by sex and age. According to a Canadian study, the odds of depression were lower for women (vs men) who were single, widowed, or separated.^28^ A Brazilian study using nationwide data found no evidence of an association between marital status and depression in the general population,^29^ although only conventional marriages were considered. Nevertheless, a study using American and Japanese data found that unmarried people had more depressive symptoms.^30^

Further research about such associations is needed among the working population, since studies on the relationship between occupational and sociodemographic (marital status, sex, and age) factors and the development of CMD are scarce in the Brazilian literature. Our study is a step towards clarifying these relationships.

The prevalence of CMD in our sample was close to that of the general Brazilian population,^6,7^ which has a high rate of work absenteeism due to CMD symptoms. CMD affect work capacity in terms of both work atendance and productivity. Because depression and anxiety account for 8% of all years lived with disability worldwide,^24^ health services must develop diagnostic and follow-up strategies to maintain the economic health of society.

### OCCUPATIONAL ASPECTS

Although the investigated occupational variables lost significance after the multivariate regression analysis, it is important to discuss other aspects of work, such as psychosocial factors, which are frequently correlated with the development of pathologies. Further studies are needed to achieve this goal.

### LIMITATIONS

This study has some limitations worth noting. First, due to its cross-sectional design, it was impossible to determine which factors initially emerged. No specific instrument was used to measure pain. Moreover, we could not assess workers who were absent from work, and we could not perform a complete analysis of psychosocial factors.

## CONCLUSIONS

Our study helped identify factors related to CMD among workers in a Brazilian service sector. Reducing activities that increase body pain may help reduce CMD symptoms. We point out the importance of corporate occupational health services, as well as primary health care services, which can perform epidemiological screening and identify workers with initial symptoms and undiagnosed CMD, as well as investigate factors that trigger or aggravate CMD, so that adequate treatment can be provided, thus reducing absenteeism and disability rates, which can result in individual, collective, and corporate benefits.

## References

[1] World Health Organization (2017). Depression and other common mental disorders: global health estimates
[Internet].

[2] Ribeiro HKP, Santos JDM, Silva MG, Medeiro FDA, Fernandes MA (2019). Transtornos de ansiedade como causa de afastamentos
laborais. Rev Bras Saude Ocup.

[3] The Lancet Global Health (2020). Mental health matters. Lancet Glob Health.

[4] Stansfeld S, Clark C, Bebbington PE, King M, Jenkins R, Hinchliffe S, McManus S, Bebbington PE, Jenkins R, Brugha T (2016). Mental health and wellbeing in England: Adult Psychiatric Morbidity Survey
2014.

[5] Steel Z, Marnane C, Iranpour C, Chey T, Jackson JW, Patel V (2014). The global prevalence of common mental disorders: a systematic review and
meta-analysis 1980–2013. Int J Epidemiol.

[6] Costa CO, Branco JC, Vieira IS, Souza LDM, Silva RA (2019). Prevalência de ansiedade e fatores associados em
adultos. J Bras Psiquiatr.

[7] Melo APS, Bonadiman CSC, Andrade FM, Pinheiro PC, Malta DC (2023). Depression Screening in a population-based study: Brazilian National Health
Survey 2019. Cienc Saude Colet.

[8] Tsuang MT, Bar JL, Stone WS, Faraone SV (2004). Gene-environment interactions in mental disorders. World Psychiatry.

[9] Galea S, Uddin M, Koenen K (2011). The urban environment and mental disorders: epigenetic
links. Epigenetics.

[10] Arango C, Dragioti E, Solmi M, Cortese S, Domschke K, Murray RM (2021). Risk and protective factors for mental disorders beyond genetics: an
evidence-based atlas. World Psychiatry.

[11] Hertenstein E, Feige B, Gmeiner T, Kienzler C, Spiegelhalder K, Johann A (2019). Insomnia as a predictor of mental disorders: a systematic review and
meta-analysis. Sleep Med Rev.

[12] Pereira FG, França MH, Paiva MCA, Andrade LH, Viana MC (2017). Prevalence and clinical profile of chronic pain and its association with
mental disorders. Rev Saude Publica.

[13] Ohayon MM, Schatzberg AF (2003). Using chronic pain to predict depressive morbidity in the general
population. Arch Gen Psychiatry.

[14] Meucci RD, Fassa AG, Faria NM (2015). Prevalence of chronic low back pain: systematic review. Rev Saude Publica.

[15] Tsuji T, Matsudaira K, Sato H, Vietri J (2016). The impact of depression among chronic low back pain patients in
Japan. BMC Musculoskelet Disord.

[16] Mok LC, Lee IFK (2008). Anxiety, depression and pain intensity in patients with low back pain who
are admitted to acute care hospitals. J Clin Nurs.

[17] Haeffner R, Kalinke LP, Felli VEA, Mantovani MF, Consonni D, Sarquis LMM (2018). Absenteeism due to musculoskeletal disorders in Brazilian workers:
thousands days missed at work. Rev Bras Epidemiol.

[18] Vignola RCB, Tucci AM (2014). Adaptation and validation of the depression, anxiety and stress scale
(DASS) to Brazilian Portuguese. J Affect Disord.

[19] Vignola RCB (2013). Escala de depressão, ansiedade e estresse (DASS): adaptação e
validação para o português do Brasil.

[20] Liu Y, Zhao J, Fan X, Guo W (2019). Dysfunction in serotonergic and noradrenergic systems and somatic symptoms
in psychiatric disorders. Front Psychiatry.

[21] Sheng J, Liu S, Wang Y, Cui R, Zhang X (2017). The link between depression and chronic pain: neural mechanisms in the
brain. Neural Plast.

[22] Rocha MRA, Marin MJS, Seda JM (2019). Fatores associados ao transtorno mental comum em trabalhadores de
serviço de limpeza hospitalar. Rev Rene.

[23] Araujo JA, Campos MR, Santos MVF, Gonçalves DA, Mari JJ, Tófoli LF (2018). Dor lombar e transtornos mentais comuns na Estratégia Saúde
da Família: uma associação pouco reconhecida. Rev Bras Med Fam Comunidade.

[24] Daré LO, Bruand PE, Gérard D, Marin B, Lameyre V, Boumédiène F (2019). Co-morbidities of mental disorders and chronic physical diseases in
developing and emerging countries: a meta-analysis. BMC Public Health.

[25] Fluharty M, Taylor AE, Grabski M, Munafò MR (2017). The association of cigarette smoking with depression and anxiety: a
systematic review. Nicotine Tob Res.

[26] Cuijpers P, Smit F, ten Have M, de Graaf R (2007). Smoking is associated with first-ever incidence of mental disorders: a
prospective population-based study. Addiction.

[27] Munafò MR, Araya R (2010). Cigarette smoking and depression: a question of causation. Br J Psychiatry.

[28] Bulloch AG, Williams JV, Lavorato DH, Patten SB (2017). The depression and marital status relationship is modified by both age and
gender. J Affect Disord.

[29] Munhoz TN, Nunes BP, Wehrmeister FC, Santos IS, Matijasevich A (2016). A nationwide population-based study of depression in Brazil. J Affect Disord.

[30] Inaba A, Thoits PA, Ueno K, Gove WR, Evenson RJ, Sloan M (2005). Depression in the United States and Japan: gender, marital status, and SES
patterns. Soc Sci Med.

